# [Retracted] Fe_3_O_4_-solamargine induces apoptosis and inhibits metastasis of pancreatic cancer cells

**DOI:** 10.3892/ijo.2024.5657

**Published:** 2024-05-20

**Authors:** Xiaodong Xie, Xiuming Zhang, Jun Chen, Xun Tang, Meiqin Wang, Lei Zhang, Zhen Guo, Wenrong Shen

Int J Oncol 54: 905-915, 2019; DOI: 10.3892/ijo.2018.4637

Following the publication of the above article, a concerned reader drew to the Editor's attention that certain of the Transwell invasion assay data shown in Fig. 5B on p. 911 were strikingly similar to data that had appeared in a previously published paper written by different authors at a different research institute.

In view of the fact that certain of the data in the above article had already appeared in a previously published paper, the Editor of *International Journal of Oncology* has decided that this paper should be retracted from the publication. The authors were asked for an explanation to account for these concerns, but the Editorial Office did not receive a reply. The Editor apologizes to the readership for any inconvenience caused.

